# QuickStats: Age-Adjusted Death Rates,* by Race/Ethnicity^†^ — National Vital Statistics System, United States, 2014–2015

**DOI:** 10.15585/mmwr.mm6613a6

**Published:** 2017-04-07

**Figure:**
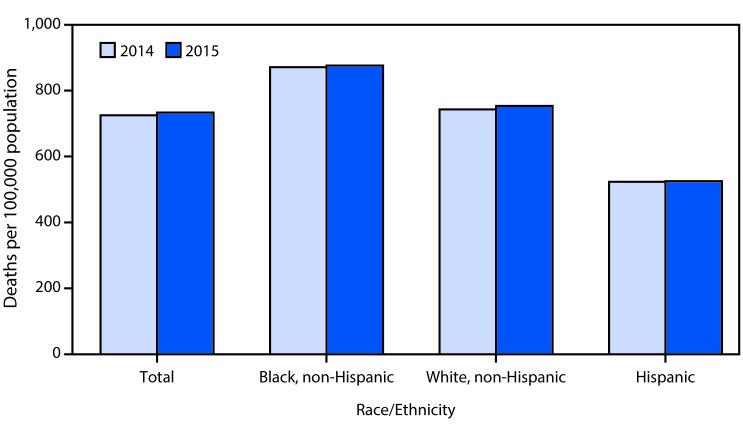
From 2014 to 2015, the age-adjusted death rate for the total U.S. population increased 1.2% from 724.6 to 733.1 per 100,000 population. The rate increased 0.6% from 870.7 to 876.1 for non-Hispanic blacks and 1.4% from 742.8 to 753.2 for non-Hispanic whites. The rate for Hispanic persons did not change significantly. The highest rate was recorded for the non-Hispanic black population, followed by the non-Hispanic white and Hispanic populations.

**Source:** National Vital Statistics System. Underlying cause of death data, 2014–2015. https://wonder.cdc.gov/ucd-icd10.html.

